# Surgical Instruments

**Published:** 1850-11

**Authors:** 


### SURGICAL INSTRUMENTS.

The attention of the reader is asked to the advertisement of Mr. Brown, surgical instrument maker. Mr. Brown has resided in this city a number of years, and we take pleasure in commending him as a faithful, skilful, and excellent workman. He has made a number of instruments for the writer, and they proved themselves fully equal to those of the most celebrated manufacturers. B.

